# Vision-Based On-Road Nighttime Vehicle Detection and Tracking Using Improved HOG Features

**DOI:** 10.3390/s24051590

**Published:** 2024-02-29

**Authors:** Li Zhang, Weiyue Xu, Cong Shen, Yingping Huang

**Affiliations:** 1 School of Optical-Electrical and Computer Engineering, University of Shanghai for Science and Technology, Shanghai 200093, China; zhangli@xyl.cn; 2 Changzhou Xingyu Automotive Lighting System Co., Ltd., 182 Qinling Road, Changzhou 213000, China; 3 School of Mechanical Engineering & Rail Transit, Changzhou University, Changzhou 213164, China

**Keywords:** monocular vision, vehicle detection, histograms of oriented gradients, non-maximum suppression, Kalman filter

## Abstract

The lack of discernible vehicle contour features in low-light conditions poses a formidable challenge for nighttime vehicle detection under hardware cost constraints. Addressing this issue, an enhanced histogram of oriented gradients (HOGs) approach is introduced to extract relevant vehicle features. Initially, vehicle lights are extracted using a combination of background illumination removal and a saliency model. Subsequently, these lights are integrated with a template-based approach to delineate regions containing potential vehicles. In the next step, the fusion of superpixel and HOG (S-HOG) features within these regions is performed, and the support vector machine (SVM) is employed for classification. A non-maximum suppression (NMS) method is applied to eliminate overlapping areas, incorporating the fusion of vertical histograms of symmetrical features of oriented gradients (V-HOGs). Finally, the Kalman filter is utilized for tracking candidate vehicles over time. Experimental results demonstrate a significant improvement in the accuracy of vehicle recognition in nighttime scenarios with the proposed method.

## 1. Introduction

As one of the basic directions of automatic driving technology, vehicle detection provides technical support for the follow-up anti-collision early warning system and has become the current research hotspot. Numerous studies on vehicle detection using monocular vision primarily concentrate on identifying vehicles during daylight hours [1,2]. Despite the traffic flow at night being much lower than during the daytime, insufficient illumination, complex road conditions, and the inapparent car contour lead to 42% of accidents occurring at nighttime [3]. The rear lights are the most significant feature of moving vehicles at night [4]. In contrast, features such as symmetry, color, shadows, and edges, usually used in daytime, are not prominent [5,6,7].

Currently, some research on vehicle detection at nighttime uses a template-based method [8,9], which mainly depends on the shape similarity and coordinate orientation features of vehicle light. Nevertheless, establishing appropriate thresholds for similarity and coordinates poses a challenge, leading to potential false positives or missed detections and consequently compromising the system’s robustness. Some scholars also proposed machine learning-based methods, mainly using features such as Haar, histogram of oriented gradient (HOG), or deformable parts model (DPM) [10,11] for classification. Song [12] used the Gaussian mixture model of spatial relations to detect daytime and nighttime vehicles in complex urban scenes. Chien et al. [13] proposed an improved multi-scale retinal image enhancement algorithm, which utilizes a two-level classifier based on a bag of features (BoF) and a convolutional neural network (CNN) to reduce false positives. Kuang et al. [14] proposed using a bionic night image enhancement method and support vector machines (SVM) classifier based on the fractional feature fusion method to achieve vehicle detection. Satzoda and Trivedi [10] used the adaptive boosting (AdaBoost) classifier based on Haar features to generate the boundary box of the vehicle for the input gray image; they used the calibration, color, and other features to verify the vehicle area producing a dataset for night vehicle detection. The vehicle feature information is always incomplete, influenced by insufficient illumination and light reflection at night. Dai et al. [15] proposed a method for night vehicle detection using grayscale division and Hough transform strategy and obtained a high detection rate for night vehicle detection and low false alarms.

Machine learning algorithms exhibit good generalization to unseen data. However, most machine learning methods only focus on the lamp information, which causes substantial difficulties for vehicle detection when the vehicle lamp is similar to other light sources on the road. Then, the deep learning methods abounded, for example, the one-stage object detection models (you only look once, YOLO, and single shot multi-box detector, SSD) [16,17] and two-stage object detection models (region CNN (R-CNN), fast-RCNN, and faster R-CNN) [18,19,20]. Gao et al. [21] used vision and lidar data fusion to detect vehicles. Mo et al. [22] proposed a neural network iteratively trained with pseudo labels based on the highlighted area of cars, using a hierarchical training strategy to solve the problem of significant intra-class differences and small inter-class differences between vehicles. Zhang and Zhu [23] proposed an improved multi-source vehicle detection algorithm based on YOLOv3, which can detect vehicles in low-resolution and low-contrast aerial infrared images. The one-stage models performed well in detection speed, and the two-stage models showed good accuracy in vehicle detection [24]. However, studies are also needed to explore the advantages of machine learning through low-cost labeling of images, computational resources, and interpretability requirements. There are scenarios where traditional machine learning methods might have advantages over deep learning in image recognition. (1) Data efficiency: machine learning methods can perform well with smaller datasets. Deep learning often requires large amounts of labeled data to generalize effectively, and it may struggle when labeled examples are limited. (2) Interpretability: machine learning models are generally more interpretable than deep learning models. Understanding the decision-making process of a machine learning algorithm can be critical, especially in sensitive applications or industries. (3) Computational resources: deep learning models and intense neural networks typically require significant computational resources (such as powerful GPUs or TPUs) for training. In contrast, some machine learning algorithms can be trained on less powerful hardware. (4) Feature engineering: machine learning often requires manual feature engineering, where domain knowledge is used to extract relevant features from the data. In certain situations, carefully crafted features may outperform automatically learned features in deep learning. (5) Computational speed: for some tasks, machine learning models might provide faster inference times than deep learning models, especially in critical real-time processing situations. (6) Resource constraints: machine learning models might be more practical than deploying resource-intensive deep learning models in applications with limited computational resources, such as edge devices or IoT devices. Therefore, deep learning methods suffer the properties of data-driven and a great deal of data labeling. In practical vehicle identification problems, it is difficult to ensure a substantial and wide variety of objects [25].

**Figure 1:**
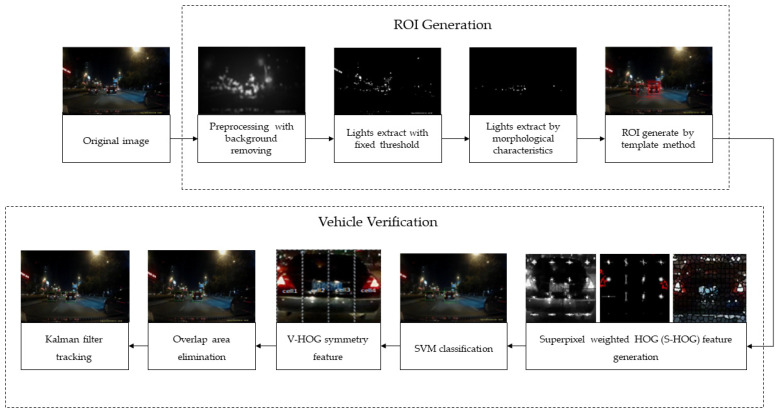
Flowchart of the proposed method for vehicle detection at night.

Regarding the hardware cost problems mentioned above, a vehicle nighttime detection method based on the saliency model and improved saliency histogram of oriented gradient (S-HOG) features is proposed in this paper. The flow chart is shown in Figure 1. One can mainly be divided into two steps: region of interest (ROI) generation and vehicle verification. (1) The removal of background illumination (saliency segmentation-based and template-based methods) was applied in the object proposal method to create the ROI. (2) The region-based superpixel and HOG features of grey and red images were combined for SVM classification. Then, the non-maximum suppression method fusion of S-HOG symmetric features is used to eliminate the overlapped areas and improve vehicle detection accuracy. Finally, the Kalman filter is utilized to track the candidate vehicle.

## 2. Proposed Method

### 2.1. Object Proposal Generation Method

#### 2.1.1. Background Light Removing Based on Saliency Segmentation

**Figure 2:**
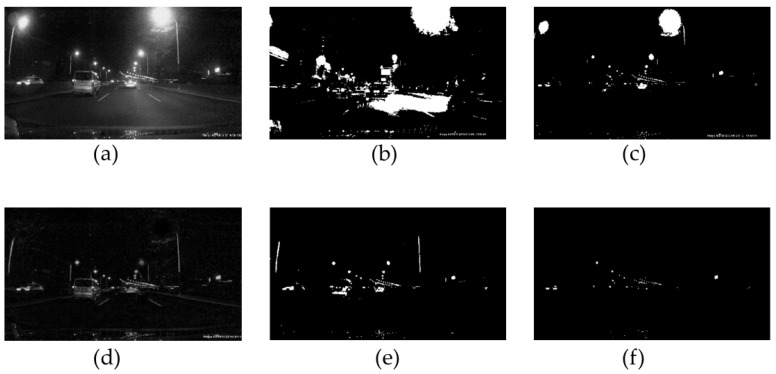
Highlight extract: (**a**) gray image; (**b**) traditional Otsu; (**c**) fixed threshold method; (**d**) background illumination removal; (**e**) traditional Otsu based on saliency model; and (**f**) fixed threshold based on saliency model.

In a complex illumination environment, significantly interfered with by vehicle reflection and streetlamp illumination, the performance of global vehicle binarization is poor. The traditional Otsu method cannot separate small bright spots from an image. In the literature [26], a fixed threshold method is proposed to obtain a fixed threshold for segmentation by the brightness characteristics of lights. As shown in Figure 2c, although this method performs better than the traditional Otsu method illustrated in Figure 2b, it still lacks robustness.

**Figure 3:**
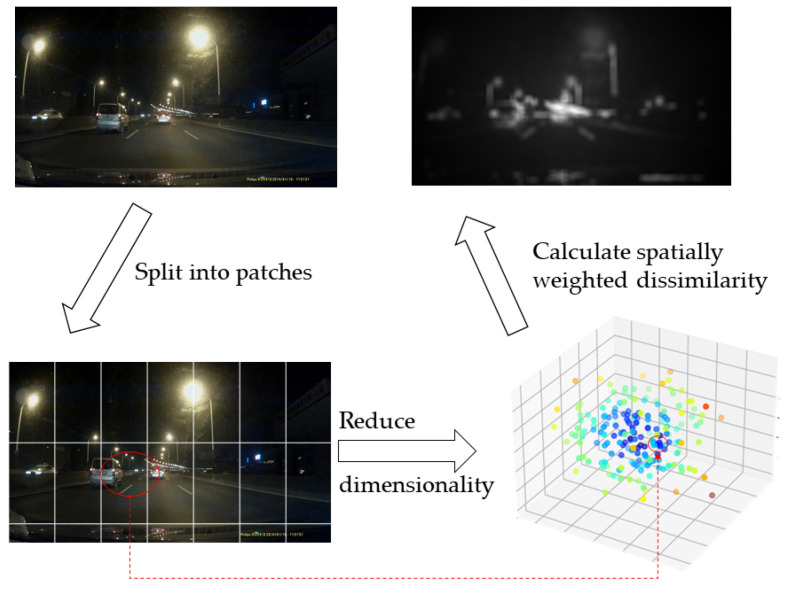
Saliency model generation.

To solve the problems above, we proposed a background light removal method based on saliency detection (Figure 3). A good saliency detection model should satisfy at least three criteria: (1) good detection: the probability of missing real salient regions and falsely marking the background as a salient region should be low, (2) high resolution: saliency maps should have a high or full resolution to locate salient objects and retain original image information accurately, and (3) computational efficiency: as front-ends to other complex processes, these models should detect salient regions quickly.

There are two main classifications of saliency model detection: patch-based and region-based. Patch-based detection models include fast and efficient saliency (FES), self-resemblance (SER), saliency segmentation (SEG), and spatially weighted dissimilarity (SWD). These methods suffer from two drawbacks: high-contrast edges usually stand out rather than salient objects, and the boundaries of salient objects are not correctly preserved. To overcome these problems, some methods propose computing saliency based on cues. Two main advantages: the number of regions is much less than the number of blocks, which implies the potential to develop efficient and fast algorithms; more informative features can be extracted from regions, leading to better performance. Region-based saliency models include covariances (COV), dense and sparse reconstruction (DSR), Markov chain (MC), and robust background detection (RBD). The main advantages of region-based saliency models: (1) complementary priors are employed to improve overall performance, which is the main advantage; (2) regions provide more complex cues (such as color histograms) compared to pixels and color patches to better capture the salient objects of the scene; (3) since the number of regions in the image is much smaller than the number of pixels, the computational saliency at the region level can significantly reduce the computational cost when generating full-resolution saliency maps. Additionally, there are some other state-of-the-art models based on context, estimation, and graph, such as context-aware (CA), graph-regularized (GR), and spectral residual (SR).

Compare Figure 2a with Figure 2d; it shows that the method we proposed removes the complex illumination. Figure 2e is a traditional Otsu method based on Figure 2d, and Figure 2f is a proposed method based on Figure 2d; it is evident that the proposed method performs better than other methods.

#### 2.1.2. Segmentation Method Based on Template

Although the most recent research uses a template-based method, due to the angle and overexposure, using the similarities of light shapes and coordinate features can easily cause false detection. The two lights of one vehicle are often horizontally aligned on a captured image, so we only use light coordinate features weighted by a saliency map to generate ROI.

The ROI generate stage uses the following characteristics of lights:

The lateral distance $D_{l}$ between two lights on the same vehicle is shorter than the maximum of the vehicle $Th_{l}$:

(1) $$\begin{array}{c} D_{l}\left(L_{1},L_{2}\right)<Th_{l} \end{array}$$

Two lights on the same vehicle are basically at the same horizontal position:

(2) $$\begin{array}{c} \frac{D_{h}\left(L_{1},L_{2}\right)}{D_{l}\left(L_{1},L_{2}\right)}<Th_{hl} \end{array}$$

Although only using coordinate features of lights could cause false detection, it can avoid missing detection and facilitate subsequent verification phase operations.

### 2.2. Hypotheses Verification

Saliency areas generated by coordinate features may contain some non-vehicle regions requiring further verification. Due to the complex nighttime environment, the traditional method using the similarity of light shapes will lead to false detection [27]. Since the HOG feature has the advantage of robustness to lighting conditions, invariance to geometric transformations, effectiveness in cluttered backgrounds, dimensionality reduction, compatibility with machine learning algorithms, high detection accuracy, and adaptability to real-time applications [28], it is often used in vehicle detection. However, due to the time-consuming and low noise immunity of the HOG feature and the lack of vehicle contour pieces of information at night, the effect of using contour features for classification does not perform as well as in daytime [22]. We propose S-HOG features combining superpixel and HOG by grey and red images, which reflect the vehicle contour and lamp contour, respectively, and then use SVM for classification to improve the accuracy of vehicle recognition.

#### 2.2.1. S-HOG Feature Extraction

**Figure 4:**
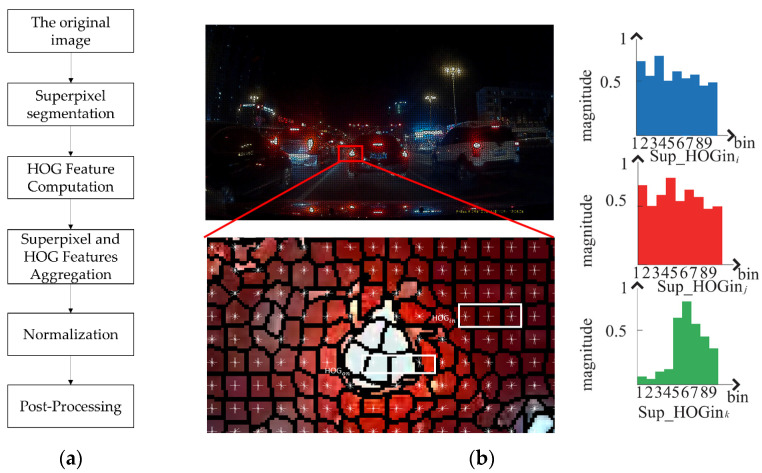
Superpixel and HOG aggregation: (**a**) aggregation flowchart and (**b**) superpixel weighted HOG feature generation.

A region-based superpixel method was fused in the HOG feature. Simple linear iterative clustering zero (SLIC0) superpixel was used in the pre-processing method to weigh each pixel in HOG feature generation without compactness factor as input and calculated automatically. The fusion approach aims to improve spatial coherence, handle variations in vehicle orientation, provide adaptive feature extraction, reduce noise, enhance discriminative power, utilize contextual information, and create a more comprehensive and robust feature representation for accurate vehicle recognition in diverse and challenging conditions (Figure 4a). The good performance of S-HOG relies on its ability to exploit the synergies between superpixels and HOG, leading to improved performance over using either technique in isolation. The sketch map is shown in Figure 4. The automatic value adapts to the content of the superpixel, leading to SLIC0 being better suited for texture and non-texture regions, the advantages of which come at the cost of slightly poor boundary adherence to regions. There is a small computational overhead and memory overhead to original simple linear iterative clustering (SLIC), which outweigh the small disadvantages for most applications of superpixels.

This paragraph adopts a method that combines S-HOG features of grey and red images. As mentioned in [29], the middle of rear lights often highlights white surrounding the red area. Firstly, change the image from RGB to HSV, the grey image is obtained to the V channel, and the red image is obtained in Table 1, and HOG features such as $H_{Gray}$ and $H_{Red}$ are extracted, respectively.

The grey and red channel images are normalized to 32 × 32 pixels, 8 × 8 pixels are selected as one HOG cell, and each block contains four cells. Since the edge gradients differ by 180° can be regarded as the same direction, the gradient direction of pixels is evenly divided into nine histogram channels, and the HOG feature vector of each training sample is 324. The 324-dimensional feature of the grey image is concatenated with the 324-dimensional feature of the red image to obtain a 648-dimensional vector for vehicle verification.

#### 2.2.2. SVM Training

**Figure 5:**
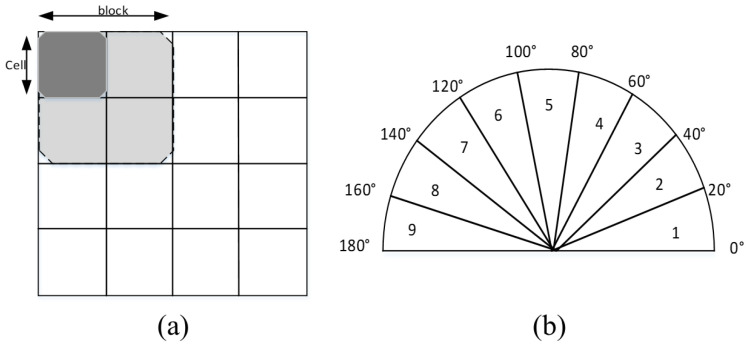
HOG features: (**a**) cells and blocks and (**b**) bins.

The HOG feature with SVM classification is widely used in vehicle and pedestrian detection [7,30]. This paper uses the radial basis function (RBF) to construct the optimal hyper-plane. Samples of 5705 positive and 3669 negative are selected for training to obtain the classifier. The positive samples include cars, vans, buses, and trucks, and the negative samples include traffic signs, road guardrails, streetlights, and billboards. As Figure 5b shows, this method performs better than the traditional HOG feature but also is required to remove overlapping.

#### 2.2.3. Overlapped Area Removal

**Figure 6:**
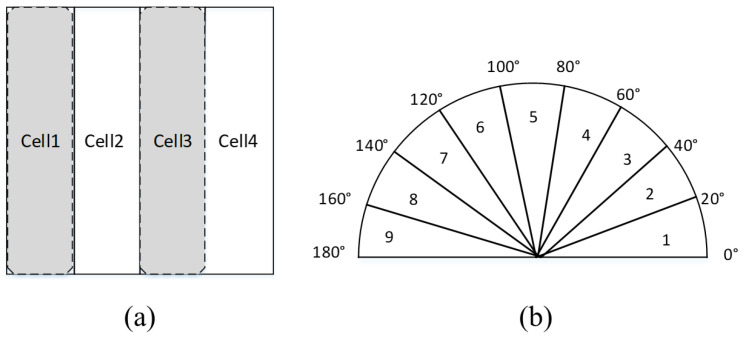
V-HOG features: (**a**) cells and (**b**) bins.

The principle of non-maximum suppression is to select the most confident bounding boxes while suppressing overlapping or redundant detections. NMS is chosen for its ability to improve precision, recall, and localization accuracy in object detection tasks, making it a crucial component in many state-of-the-art detection systems (such as vehicle detection) [31]. Non-maximum suppression (NMS) uses the highest score obtained by the classifier to suppress low-scoring windows and eliminate redundant detection areas. Due to the symmetry of vehicles, it is proposed to use the V-HOG feature [30] to calculate the score. Figure 6 shows that the V-HOG feature divides the image into four vertical parts; each part is a HOG cell with nine bins.

**Figure 7:**
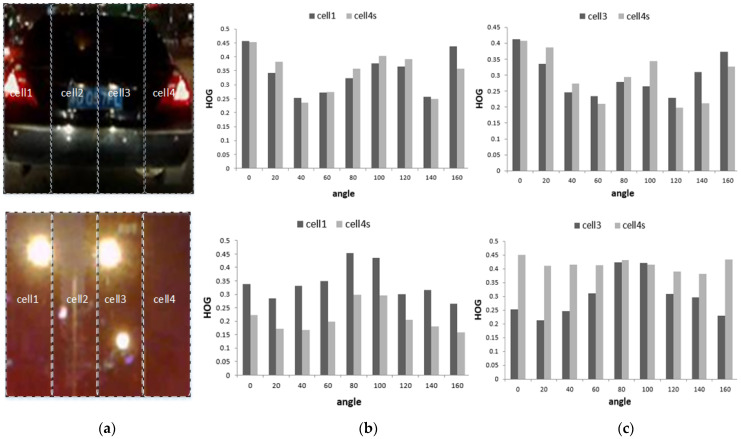
V-HOG symmetry: (**a**) ROI image; (**b**) cell1 and cell4s HOG symmetry; and (**c**) cell2 and cell3s HOG symmetry.

As Figure 7 shows, since the vehicle is more symmetrical than in other areas, so if there is a vehicle in the ROI area, cell1 and cell2 are symmetrical to cell3s and cell4s, and the definition of these four cells is in Equations (3)–(6):

(3) $$\begin{array}{c} cell1=\left[{bin}_{11}\;{bin}_{12}\;{bin}_{13}\;{bin}_{14}\;{bin}_{15}\;{bin}_{16}\;{bin}_{17}\;{bin}_{18}\;{bin}_{19}\right] \end{array}$$

(4) $$\begin{array}{c} cell2=\left[{bin}_{21}\;{bin}_{22}\;{bin}_{23}\;{bin}_{24}\;{bin}_{25}\;{bin}_{26}\;{bin}_{27}\;{bin}_{28}\;{bin}_{29}\right] \end{array}$$

(5) $$\begin{array}{c} cell3s=\left[{bin}_{39}\;{bin}_{38}\;{bin}_{37}\;{bin}_{36}\;{bin}_{35}\;{bin}_{34}\;{bin}_{33}\;{bin}_{32}\;{bin}_{31}\right] \end{array}$$

(6) $$\begin{array}{c} cell4s=\left[{bin}_{49}\;{bin}_{48}\;{bin}_{47}\;{bin}_{46}\;{bin}_{45}\;{bin}_{44}\;{bin}_{43}\;{bin}_{42}\;{bin}_{41}\right] \end{array}$$

The similarity value calculation based on Euclidean distance is a method with a high usage rate. As Equations (7) and (8), the similarity value $sin\left(x,y\right)$ is between [0,1]. The $x_{i}$ means bins in cell 1 and cell 2, $y_{i}$ means bins in cell3s and cell4s, $d\left(x,y\right)$ means the distance between cells. The higher the value of the $sin\left(x,y\right)$, the higher the similarity.

(7) $$d\left(x,y\right)=\sqrt{\Sigma {\left(x_{i}-y_{i}\right)}^{2}}$$

(8) $$sin\left(x,y\right)=\frac{1}{1+d\left(x,y\right)}$$

The similarity value is used as the score of NMS, and the overlap degree is set to 0.3 to remove the overlap area, as shown in Figure 7c.

#### 2.2.4. Vehicle Tracking

The vehicle movement is continuous, so the position and size changes of the same car in sequence images are also continuous. Prediction and tracking of vehicle targets can improve the accuracy of vehicle detection.

In the video, the displacement of the same vehicle between two consecutive frames is relatively minor, and the vehicle’s motion approximates uniform linear movement. In variable-speed linear motion, turning, parking, and similar conditions, the small-time interval between frames mitigates the approximation error associated with uniform rectilinear motion, treating these errors as system noise. Furthermore, a corresponding relationship exists between the position and size of the vehicle in the image. Therefore, it can be assumed that the size change of the vehicle in sequence images is approximately uniform, and the approximate error can be considered system noise. The system state equation can be expressed as follows:

(9) $$\begin{array}{c} X_{k}=AX_{k-1}+Bu_{k}+w_{k} \end{array}$$

(10) $$\begin{array}{c} X={\left[x,y,w,h,\Delta x,\Delta y\right]}^{T} \end{array}$$

(11) $$\begin{array}{c} A=\left[\begin{array}{c} 1\;0\;0\;0\;1\;0\\ 0\;1\;0\;0\;0\;1\\ 0\;0\;1\;0\;0\;0\\ 0\;0\;0\;1\;0\;0\\ 0\;0\;0\;0\;1\;0\\ 0\;0\;0\;0\;0\;1 \end{array}\right] \end{array}$$

(12) $$\begin{array}{c} B={\left[0,0,0,0,0,0\right]}^{T} \end{array}$$

X is the system state vector, (x, y) is the image coordinate in the midpoint of the vehicle lower boundary, w and h are the width and height of the target in the image coordinate system. A is the system status transfer matrix. The system measurement equation is:

(13) $$\begin{array}{c} Z_{k}=HX_{k}+v_{k} \end{array}$$

The system measurement vector is:

(14) $$\begin{array}{c} H=\left[\begin{array}{c} 1\;0\;0\;0\;0\;0\\ 0\;1\;0\;0\;0\;0\\ 0\;0\;1\;0\;0\;0\\ 0\;0\;0\;1\;0\;0\\ 0\;0\;0\;0\;1\;0\\ 0\;0\;0\;0\;0\;1 \end{array}\right] \end{array}$$

The Kalman filter can handle dynamic systems, robustness to noisy measurements, predictive-corrective nature, adaptability to changes in motion, continuous state estimation, efficient memory usage, integration with multiple sensors, predictive capability, and its well-established track record in tracking and estimation. In this article, the Kalman filter predicts the new position, width, and height and generates the expected vehicle area [32]. The prediction frame at time t matches the image recognition frame at time t+1. The maximum intersection over union (IOU) is the observed value at time t+1. If the matching fails, the final predicted value will be supposed to be the observed value. If the matching is a failure for 10 consecutive times, the tracking object will be discarded.

## 3. Results and Discussion

### 3.1. Comparison of Different Saliency Methods

**Figure 8:**
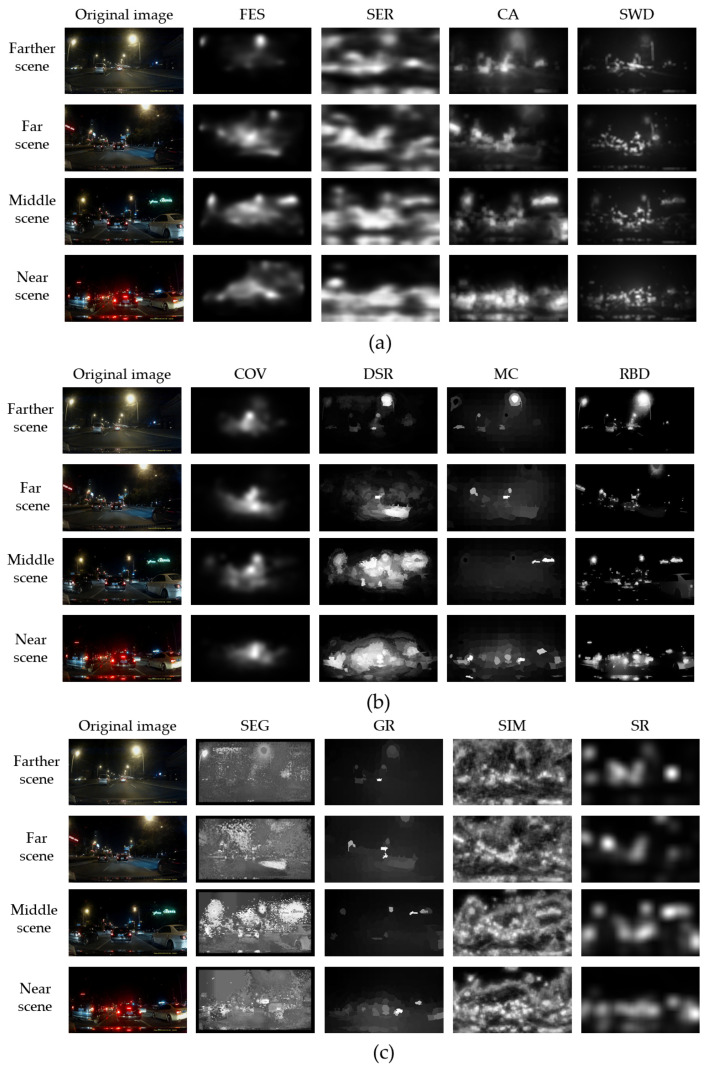
Visual comparison of 12 ROI methods: (**a**) methods based on patches; (**b**) methods based on region; and (**c**) methods based on others.

The performance of the SWD method based on patches was better than that of others for removing background illumination at night. All the streetlights were judged to be the non-saliency section, as shown in Figure 8 (column SWD). Other methods, for example, FES, SER, CA, DSR, MC, and RBD, included the environment light, e.g., streetlights and building lights. COV method also avoided the non-relevant lights, as shown in Figure 8 (column COV); however, the integrity of prospective vehicles is lower, e.g., the road near the target vehicles was identified as foreground.

The performance of saliency detection near scenes was better than the far scenes. The targets cars and non-targets are easier to confuse together in far scenes, for example, in DSR, MC, RBD, FES, and SER of the first line in Figure 8.

### 3.2. Comparison of Different Feature Extraction Methods

To verify the effectiveness of the proposed method, MATLAB R2016a is used to conduct vehicle recognition experiments in the pictures collected under real-time urban and elevated roads. The processor is Intel i5-8250, 8 G memory, with 500 and 720 × 480 image resolution.

**Figure 9:**
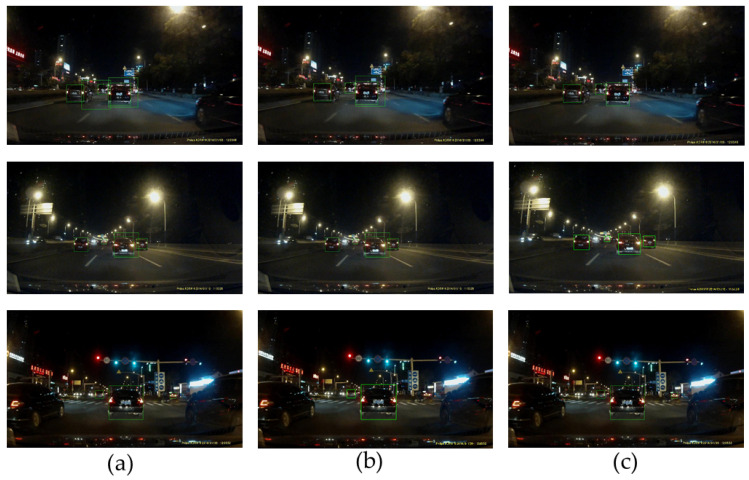
(**a**) Template method; (**b**) traditional HOG and SVM; (**c**) improved HOG and SVM. Note: the green boxes mean the detection results.

The experimental results are presented in Figure 8. Figure 9a indicates that under different road scenes, there will be misdetections when using the template method [29,33], such as color, coordinate, and shape characteristics. Figure 9b shows that some vehicles with less obvious contours will fail to be detected when using the traditional HOG feature. Figure 9c shows that the detection accuracy improved using the proposed method, especially when the vehicle contour is not apparent.

Some images of urban and highways were obtained in the night scene, and the results are shown in Table 2, using the method mentioned above. The accuracy rate and the recall rate of two key objectives are mainly based on three parameters, which are true positive (TP), false positive (FP), and false negative (FN). They are defined as Equations (15) and (16). The higher the rates, the better the results.

(15) $$\begin{array}{c} Precision=\frac{\left|TP\right|}{\left|TP\right|+\left|FP\right|} \end{array}$$

(16) $$Recall=\frac{\left|TP\right|}{\left|TP\right|+\left|FN\right|}$$

Table 2 shows the test results of the proposed and traditional methods in urban and highway scenarios. It shows that the method proposed in this paper performs better than conventional methods.

### 3.3. Deep Learning Algorithm Detection and Comparison

YOLO series object detection algorithms are object detection algorithms based on regression networks. These algorithms have obvious advantages in performance, speed, lightweight design, and flexibility. Moreover, the YOLO algorithms have been developed to derive various versions, and the development is relatively complete. They have become some of the most concerned algorithms in target detection [34,35].

The training dataset in network detection experiments includes 7800 vehicle images and 1260 headlight images with a pixel resolution of 720 × 480 from a real-time tachograph. The classification of test images and test results are shown in Table 3.

All the state-of-the-art deep learning algorithms have excellent performance in mAP, precision, and recall (above 96%). However, these deep learning networks have more train time, model size, and labeling image size than traditional machine learning methods. For example, the training time of deep learning is 19.82 times than feature engineering methods and 78.19 times in model size. The number of annotated images required is only 5% of machine learning methods than deep learning methods. The YOLO identification experiment exhibited robust detection capabilities primarily tailored to models within the dataset and similar series, indicating limited universality. Specifically, in vehicle-related projects, such as headlight recognition in engineering, a substantial amount of diverse data is essential for comprehensive training [3]. Consequently, our proposed method often outperforms in scenarios with limited data volumes. The algorithm presented in this study demonstrates a strong generalization ability, significantly enhancing classification accuracy and the overall adaptability of the final robust classifier. Moreover, it is noteworthy that deep learning models discussed in this chapter impose hardware requirements and demand extensive training periods. In contrast, the proposed model offers advantages in terms of low training time costs and significantly smaller model parameters than traditional deep learning algorithms. This makes our model well-suited for deployment in embedded devices and other hardware environments commonly encountered in general settings [36].

## 4. Conclusions

This paper proposes a nighttime vehicle detection method with hardware cost constraints, using a saliency map and superpixel weighting the grey and red channel HOG features for SVM classification and then using the NMS method to fuse S-HOG. The main advantage of the proposed method is that our method performs better in detecting vehicles with less obvious contours, especially when we use the color and S-HOG features. Additionally, the result demonstrates that this method performed better than the traditional HOG methods. Lastly, the Kalman filter was used to improve the prediction accuracy. Experimental results have shown that the proposed method performs better than conventional methods.

## Figures and Tables

**Table 1:** Red color threshold parameters.

Hue	Saturation	Value
0–30 and 150–180	10–80	190–255

**Table 2:** The test result of improved and traditional methods in urban and highway scenarios.

Scenario	Method	TP	FP	FN	Precision (%)	Recall (%)	Train Time (h)	Model Size (MB)	Image Label Size
Urban	Template	1765	469	211	79.01	89.32	0.50	0.11	500
	HOG	1676	284	300	85.51	84.82	0.51	0.15	
	Proposed	1854	147	122	92.65	93.83	0.53	0.16	
Highway	Template	472	42	28	91.83	94.40	0.49	0.11	500
	HOG	465	24	35	95.09	93.00	0.52	0.13	
	Proposed	482	28	18	94.51	96.40	0.52	0.14	

**Table 3:** YOLO algorithms detect the results of vehicles.

Model	mAP (%)	Precision (%)	Recall (%)	Param (M)	GFLOPs	FPS	Train Time (h)	Model Size (MB)	Image Label Size
YOLO v5	98.0	97.8	96.3	6.7	15.8	84.7	12.582	14.4	10,000
YOLO v6	99.5	99.4	99.0	4.0	11.8	87.7	10.950	8.7	
YOLO v7	99.8	99.0	99.0	5.7	13.1	89.3	21.829	12.3	
YOLO v8	99.5	98.9	98.7	3.0	8.1	303.03	7.202	6.3	

## Data Availability

The evaluation code and dataset are available at https://github.com/weiyuexu/Rear-Lamp-Vehicle-Detection (accessed on 1 January 2024).
